# Systemic Mastocytosis Associated with “Smoldering” Multiple Myeloma

**DOI:** 10.3390/diagnostics11010088

**Published:** 2021-01-07

**Authors:** Magda Zanelli, Stefano Ricci, Maurizio Zizzo, Francesca Sanguedolce, Federica De Giorgi, Andrea Palicelli, Giovanni Martino, Stefano Ascani

**Affiliations:** 1 Pathology Unit, Azienda Unità Sanitaria Locale-IRCCS di Reggio Emilia, 42122 Reggio Emilia, Italy; Magda.Zanelli@ausl.re.it (M.Z.); Stefano.Ricci@ausl.re.it (S.R.); Federica.DeGiorgi@ausl.re.it (F.D.G.); Andrea.Palicelli@ausl.re.it (A.P.); 2 Surgical Oncology Unit, Azienda Unità Sanitaria Locale-IRCCS di Reggio Emilia, 42122 Reggio Emilia, Italy; 3 Clinical and Experimental Medicine PhD Program, University of Modena and Reggio Emilia, 41121 Modena, Italy; 4 Pathology Unit, Azienda Ospedaliero-Universitaria-Ospedali Riuniti di Foggia, 71122 Foggia, Italy; fsanguedolce@ospedaliriunitifoggia.it; 5 Pathology Unit, Dipartimento di Medicina e Chirurgia, Università di Parma, 43125 Parma, Italy; 6 Hematology Unit, CREO, Azienda Ospedaliera di Perugia, University of Perugia, 06129 Perugia, Italy; Giovanni.Martino@unipg.it (G.M.); s.ascani@aospterni.it (S.A.); 7 Pathology Unit, Azienda Ospedaliera S. Maria di Terni, University of Perugia, 05100 Terni, Italy

**Keywords:** mastocytosis, myeloma, bone marrow

## Abstract

A 79-year-old woman presented with a long history of peripheral eosinophilia. Previous right hemicolectomy for colonic polyposis was reported. Laboratory tests were notable for mild macrocitic anaemia and eosinophilia. β2 microglobulin and serum tryptase levels were elevated. Serum immunofixation revealed IgA/kappa monoclonal protein. Bence-Jones protein was positive. Bone marrow (BM) biopsy revealed the coexistence of two neoplastic components. Cohesive clusters of bland-looking, spindle-shaped mast cells, representing 20% of marrow cellularity, were close to aggregates of mature plasma cells occupying 40% of marrow cellularity. Molecular analysis on marrow aspirate demonstrated KIT D816V mutation, TET2 mutation, monoallelic deletion of TP53/17p13 and trisomy of ATM/11q23. A bone density study revealed mild osteoporosis. Full skeletal X-rays and magnetic resonance imaging (MRI) of spine and hips showed multiple, small rarefaction areas and an old L1-L2 fracture, both ascribed to osteoporosis. The association of systemic mastocytosis (SM) and multiple myeloma (MM) is very uncommon. The coexistence of SM with MM placed our patient in the SM with associated clonal haematological non-mast-cell lineage disease (SM-AHN) subtype. Midostaurin therapy was started.

A 79-year-old woman was referred for a long history of peripheral eosinophilia. She underwent right hemicolectomy for colonic polyposis a few years before. Laboratory tests disclosed mild macrocitic anaemia (Hb 10.4 g/dL, MCV 93%) and eosinophilia (leukocytes 3360/mmc, eosinophils 30%). β2 microglobulin (3256 ng/dL; reference range 1010–1730) and serum tryptase (138 ng/L; reference range 0–11.4) were elevated. Serum immunofixation revealed IgA/kappa monoclonal protein (21 g/L). Bence-Jones protein was positive. Bone marrow (BM) biopsy revealed two neoplastic components. Low- and high-power views of haematoxylin and eosin sections showed cohesive paratrabecular aggregates of bland-looking, spindle-shaped cells (Figure 1; Figure 2 *lower part*) positive for CD117 (Figure 3), tryptase and CD25 representing 20% of marrow cellularity. Aggregates of mature plasma cells (Figure 1; Figure 2 *upper part*) positive for CD138 (Figure 4), MUM1/IRF4 and kappa light chain occupied 40% of the remaining bone marrow. Bone marrow aspirate confirmed the presence of the two neoplastic components (Figure 5). KITD816V mutation was detected by DHPLC and confirmed by Sanger sequencing; TET2 mutation was identified using DNA sequence analysis on marrow aspirate. Monoallelic deletion of TP53/17p13 and trisomy of ATM/11q23 was detected by FISH analysis on BM enriched with plasma cells using CD138+ magnetic Micro-Beads, respectively, in 39% and 94% of nuclei. RUNX1, ASXL1, SRSF2 and U2AF1 were unmutated. Osteoporosis was identified by full skeletal X-rays and MRI. The case was referred to as SM with concomitant smoldering MM.

The diagnosis of SM was proposed owing to the fulfilment of the major diagnostic criteria (multifocal, dense, compact aggregates in bone marrow and/or extra-cutaneous organs) and three of the minor criteria (KIT D816V mutation, CD25 expression, serum tryptase level exceeding 20 ng/mL) combined with the presence of a C finding (organ involvement with loss of function: intestinal mastocytosis with clinical malabsorption identified in the previous colectomy specimen) [1]. Due to the bone marrow findings, along with the absence of myeloma-defining events (frank anaemia, hypercalcemia, lytic bone lesions, renal insufficiency secondary to myeloma, recurrent bacterial infections, blood hyperviscosity, paraneoplastic neuropathy, signs of associated amyloidosis, spinal osseous lesions at MRI), our patient fitted into the diagnosis of smoldering MM.

The coexistence of SM with MM placed our patient in the SM with associated clonal haematological non-mast-cell lineage disease (SM-AHN) subtype. Midostaurin therapy (100 mg twice per day) was started.

SM-AHN, a subtype of SM recognized in the current WHO classification, is defined as meeting the criteria for SM and for an associated haematological neoplasm as a distinct entity [1]. SM-AHN represents the second most common subtype of SM, with a frequency of 10% to 20% according to large multicentric studies [2]. Over 80% of SM-AHN are associated with myeloid neoplasms and rarely with lymphoma and plasma cell dyscrasias. The association of MM with SM is very uncommon, with very few cases reported so far [3,4,5]; the neoplastic cells are believed to be derived from distinct clones; cytokines produced by mast cells are postulated to induce plasma cell proliferation [4,6].

## Figures and Tables

**Figure 1:**
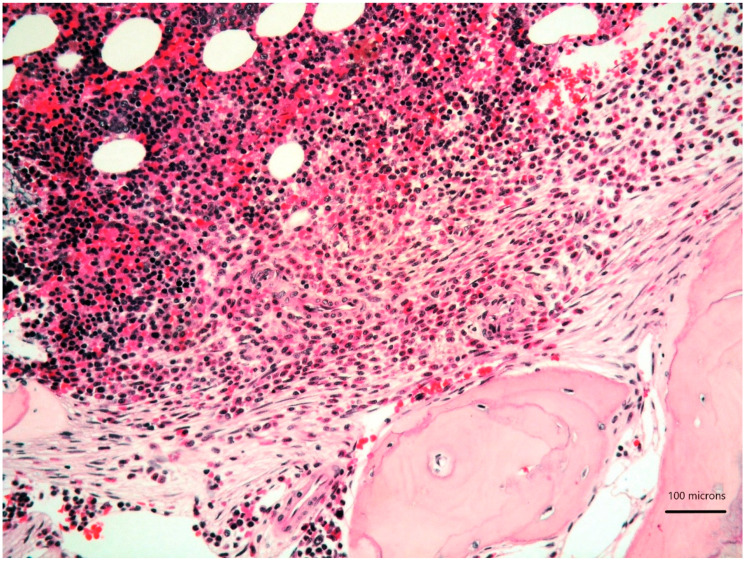
Bone marrow biopsy showing paratrabecular aggregates of spindle-shaped cells (*lower part*) close to clusters of mature plasma cells (*upper part*) (haematoxylin and eosin, 200× magnification).

**Figure 2:**
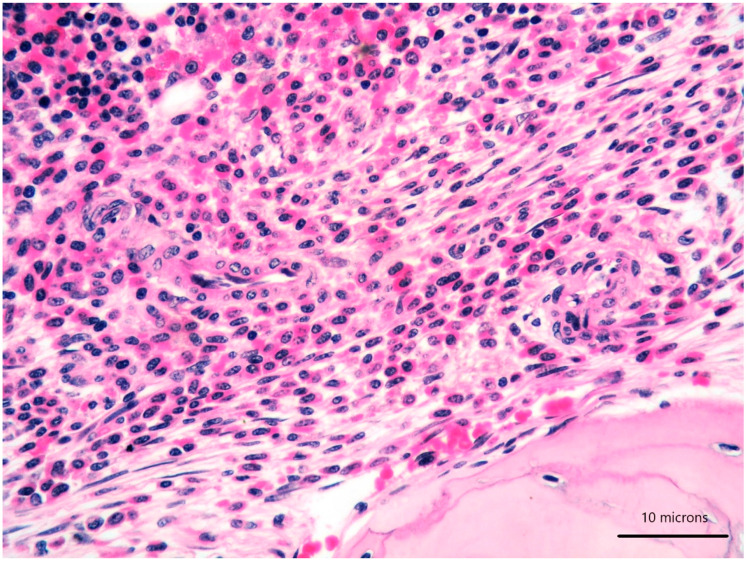
High-power view of bone marrow biopsy highlighting morphological details of bland-looking spindle cells (*lower part*) and mature plasma cells (*upper part*) (haematoxylin and eosin, 400× magnification).

**Figure 3:**
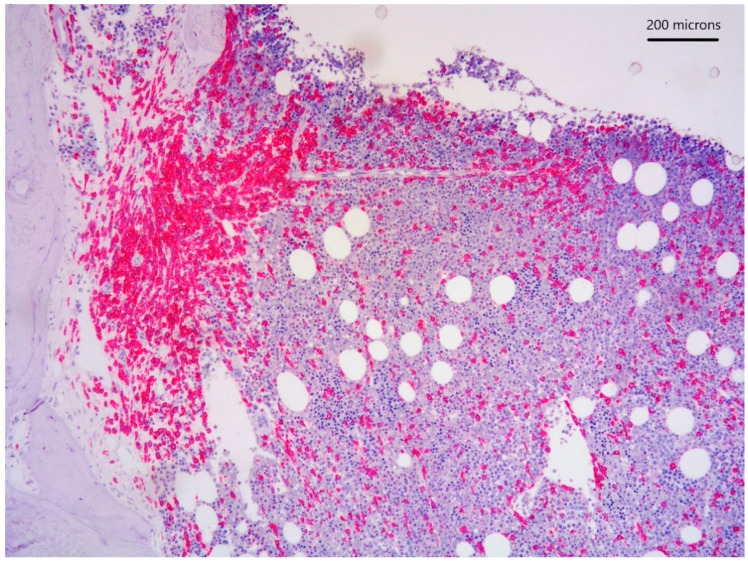
CD117 immunostain highlighting paratrabecular aggregates of spindle-shaped cells.

**Figure 4:**
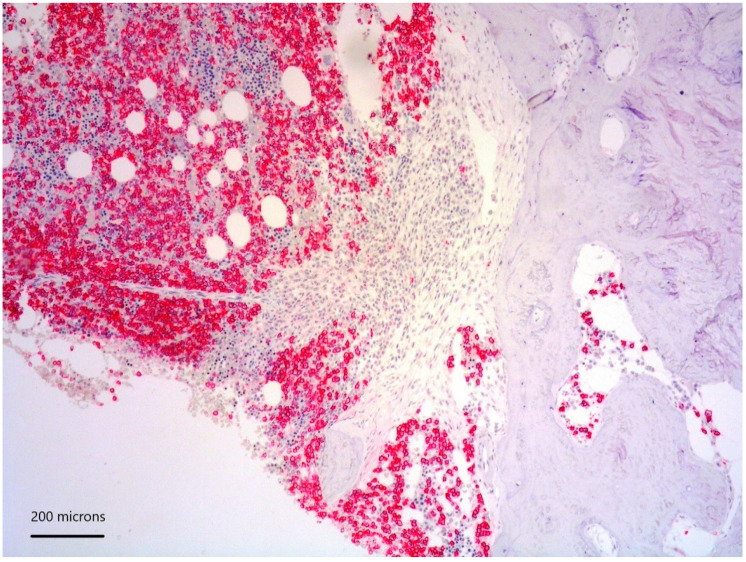
CD138 positivity of plasma cell aggregates.

**Figure 5:**
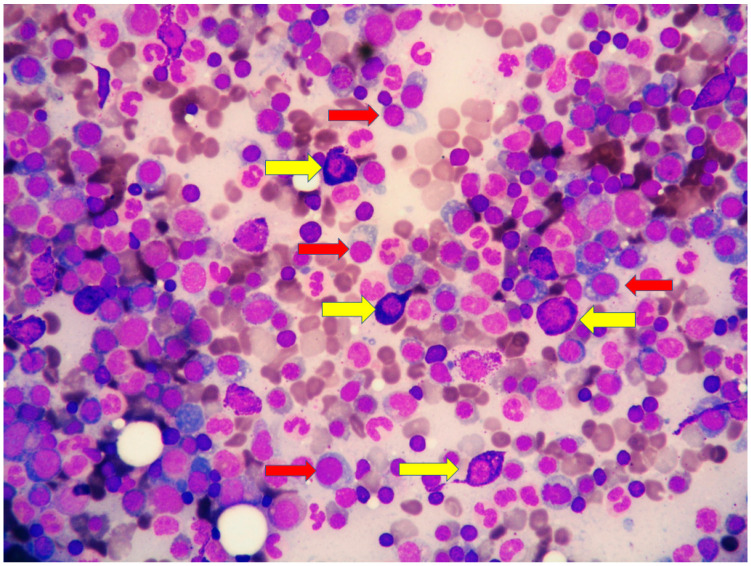
High magnification of marrow aspirate disclosing the two neoplastic components: (i) mast cells with basophilic granular cytoplasm (yellow arrows) and (ii) plasma cells with eccentric nucleus (red arrow).

## Data Availability

The data presented in this study are available on request from the corresponding author.
